# CVE-assisted Co_3_O_4_ nanoparticle-coated optical fiber sensor for acetone detection

**DOI:** 10.1038/s41598-026-47233-y

**Published:** 2026-04-06

**Authors:** Bhawani Sahu, Ramkrushna Sahu, Priyanshu Kumar Patro, Kumar Prasannajit Sahu, Anurag Das, Tusharkant Panda, Padmini Mishra, Adarsh Nigam, Pramod Martha

**Affiliations:** 1 E-YUVA BIRAC Fellow, GIET University, Gunupur, Odisha India; 2 Department of Electronics and Communication Engineering, GIET University, Gunupur, Odisha India; 3 Manipal Institute of Technology, Manipal Academy of Higher Education, Manipal, India

**Keywords:** Acetone, Cobalt oxide nanoparticle, Optical fiber sensor, Chemical vapour etching (CVE), Breath analysis, Gas sensor, Chemistry, Engineering, Materials science, Nanoscience and technology, Optics and photonics

## Abstract

In this paper, an acetone sensor based on a single-mode fiber (SMF) coated with cobalt oxide (Co$_{3}$O$_{4}$) nanoparticles is characterized. Different methods were explored for coating Co$_3$O$_{4}$ nanoparticles on SMF, and a chemical vapor etching (CVE) method was optimized to yield a functionalized SMF. The Co$_3$O$_{4}$ nanoparticles were synthesized via coprecipitation using cobalt acetate and NaOH. SEM analysis showed that CVE pretreatment created a distinct surface morphology, enabling a more uniform and homogeneous nanoparticle coating compared to the dip coating method. Extensive material characterization methods such as Fourier transform infrared (FTIR), Ultraviolet-Visible (UV-Vis) spectroscopy, dynamic Light Scattering (DLS) analysis, and scanning electron microscope (SEM), are utilized to analyze and evaluate structural stability and homogeneous distribution with optical properties of the nanoparticles and their applicability in breath analysis. A customized sensor characterization setup has been designed for acetone sensing. An optical coupler injects light into the fiber, while another directs the transmitted signal to a photodiode, which converts it into an electrical current varying with acetone concentration. This signal is processed by a microcontroller (Arduino/ESP-32). The output current increases from 0.48 $\mu$A at 0.5 ppm to 2.6 $\mu$A at 12 ppm, yielding a sensitivity of 0.18 $\mu$A/ppm with 3% non-linearity. The sensor offers a low-cost, high-performance solution for acetone detection for early diagnosis and health monitoring, supporting sustainable healthcare and innovation.

## Introduction

Volatile organic compounds (VOCs) like acetone are vital markers in medical diagnostics, industrial safety, and environmental monitoring^1,2^. As a naturally occurring VOCs and metabolic byproduct, acetone serves as a key biomarker for non-invasive diagnosis of conditions like diabetes mellitus^3^. Elevated breath acetone levels are associated with ketosis resulting from impaired glucose metabolism in diabetic patients. Sensitive and selective acetone sensors offer a promising alternative to traditional blood glucose monitoring, improving early diagnosis and patient compliance^4^. Beyond biomedical use, acetone is widely used in paint, pharmaceuticals, and polymer manufacturing industries. However, high exposure levels pose health risks including respiratory and neurological disorders^5^. Acetone, a natural metabolic byproduct, is key for non-invasive diagnosis of diseases such as diabetes mellitus^3,6^, but its widespread industrial use poses health and safety risks^5^. Continuous monitoring is essential to prevent health hazards, environmental pollution, and fire/explosion risks due to acetone’s flammability.

Several gas sensing technologies, such as gas chromatography-mass spectrometry (GC-MS), high-performance liquid chromatography (HPLC), metal oxide semiconductor field effect transistor (MOSFET)-type gas sensor, and electrochemical sensors, have been used to detect acetone^7–9^. Even though these methods have high selectivity and sensitivity, they tend to be costly and involve sophisticated instrumentation^10^. Optical fiber sensors are increasingly explored for chemical sensing due to their compactness, light weight, immunity to electromagnetic interference (EMI), remote sensing capabilities, and multiplexing potential^11^. However, achieving selectivity and sensitivity, especially for detecting hazardous or target-specific gaseous analytes, requires surface functionalization of the fiber, typically by modifying the cladding or core with selective materials^12,13^.

The performance of metal oxide semiconductor (MOS) nanoparticles in acetone sensing depends on factors such as surface area, porosity, catalytic activity, and electronic structure. While many metal oxides have shown promise, each has limitations. For instance, Magnesium oxide (MgO) offers a high surface area but lacks selectivity and requires high operating temperatures, limiting portability^14^. Titanium Dioxide (TiO_2_) demonstrates good photocatalytic activity under UV light, but its energy demand makes it less suitable for low-power applications^15^. Similarly, Tungsten Trioxide (WO_3_) shows good acetone interaction but requires UV activation and exhibits slow response and recovery times, reducing its effectiveness for real-time sensing^16^. Iron Oxide (FeO_3_) is cost-effective and easy to synthesize, but suffers from poor response times and reduced sensitivity in humid conditions. Zinc Oxide (ZnO) provides high sensitivity, especially in breath analysis, but its performance degrades under humidity^17^. Silver (Ag) nanoparticles enhance sensor response due to their conductivity and catalytic properties; however, their high cost and tendency to agglomerate limit their scalability^18^. These trade-offs underscore the need for alternative materials, such as Co_3_O_4_, which offers a more balanced profile for sensitive, selective, and practical acetone detection.

Nanostructured metal oxide semiconductors (MOS) like Tin oxide (SnO$_2$) and ZnO offer high surface area and catalytic activity for gas sensing^19^, while p-type Co$_3$O$_4$ excels with superior selectivity, stability, and room-temperature operation due to its large surface area, oxygen vacancies, and efficient charge transport^20,21^. Additionally, Co$_3$O$_4$’s compatibility with optical fiber systems enhances its potential for advanced sensing applications^22^. Numerous chemical methods can be used to prepare cobalt oxide (Co$_3$O$_4$) nanoparticles with some control over morphology, crystallinity, and particle size. Common synthesis methods for cobalt oxide nanoparticles include sol-gel and green synthesis. The sol-gel method involves heating a cobalt nitrate-ethylene glycol solution to form a gel, followed by microwave treatment and pulverization^23^, while the green method uses Azadirachta indica leaf extract as a natural reducing agent, offering a low-cost, eco-friendly alternative^24^. In the current work, the precipitation method is used for the synthesis of cobalt oxide nanoparticles because it is simple, inexpensive, and scalable. Various sizes of Co$_3$O$_4$ nanoparticles (NPs) were synthesized under controlled conditions using cobalt (II) acetate tetrahydrate ((CH$_3$COO)$_2$Co$\cdot$4H$_2$O, 99%), ethanol, and sodium hydroxide (NaOH, 99%) as the main precursors, without further purification^25^.

Acetone molecules do not have adsorption potential on their own; instead, adsorption occurs based on the interaction with the active sites on the Co$_3$O$_4$ nanoparticle surface. The layer of nanoparticle provides oxygen vacancies and cobalt cation sites that enable adsorption of acetone molecules based on dipole interaction with the carbonyl group of the acetone molecule. Simultaneously, the deposition of the Co$_3$O$_4$ layer provides additional attenuation to the optical signal, which causes a constant baseline effect with reduced transmission of the optical signal. When the sensing measurements are performed, the baseline transmission is measured before the acetone molecules interact with the nanoparticle layer, and the response is measured based on the change in transmitted optical intensity as a result of adsorption of acetone molecules on the nanoparticle layer.

**Fig. 1:**
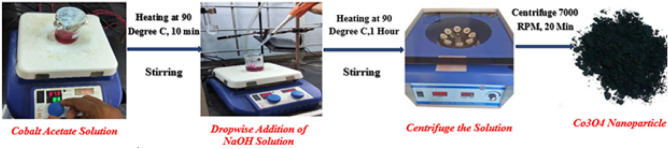
Synthesis process of the cobalt oxide nanoparticles.

Optical fiber sensors coated with Co$_3$O$_4$ benefit from enhanced light absorption, which improves signal output and detection sensitivity. Co$_3$O$_4$’s synthesis is straightforward and scalable, making it a cost-effective choice for large-scale production. Parthasarathy Srinivasan et al. developed Co$_3$O$_4$ hollow nanospheres with high sensitivity, selectivity, and stability for low-temperature acetone sensing^26^. Integrating Co$_3$O$_4$ nanoparticles onto optical fibers enables chemical-to-optical signal conversion, but achieving uniform, adherent coatings is difficult due to silica’s smooth surface^27,28^. Chemical Vapour Etching (CVE) offers improved adhesion by introducing surface roughness, though its performance remains underexplored compared to conventional methods^29^.

In this research, Co$_3$O$_4$ nanoparticles are synthesized via the precipitation method and systematically characterized for acetone sensing, followed by coating over a single-mode fiber (SMF) obtained through the chemical vapour etching (CVE) method. The results demonstrate superior sensing capabilities, confirming Co$_3$O$_4$ coated fiber as a promising material for selective acetone detection. Section II discussed the Co$_3$O$_4$ nanoparticle synthesis and its coating over the fiber. Results and discussion are demonstrated in Section III, which presents the various characterization methods, such as FTIR, UV-Vi, and SEM, carried out to observe the prepared sensor material properties, followed by the acetone sensing behaviour. Section IV concludes the paper.

**Fig. 2:**
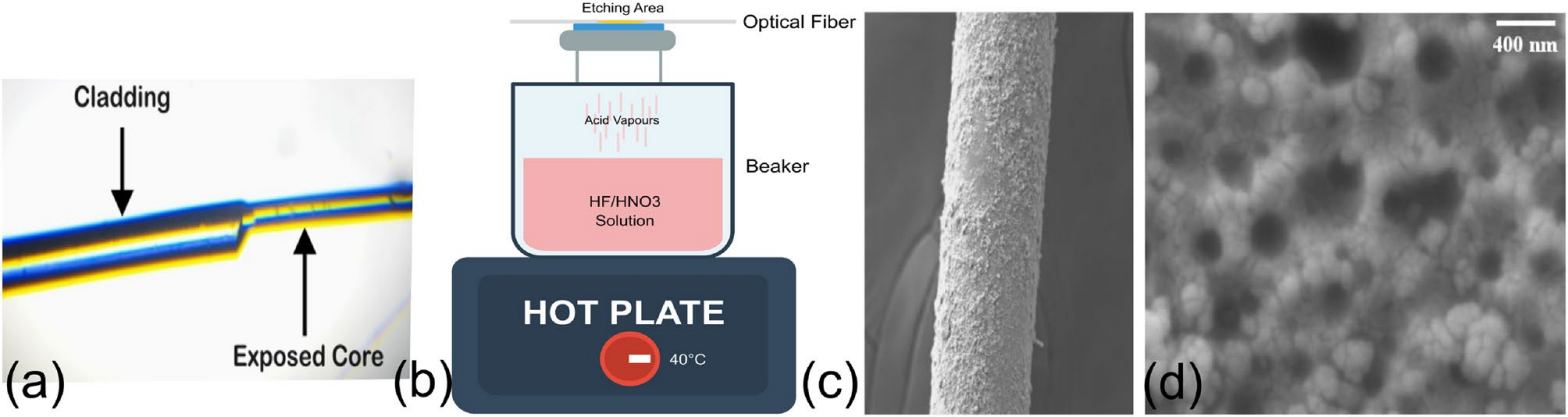
(**a**) Clad removal method exposes the core of the fiber, (**b**) Chemical Vapour Etching Method forming micro-cavities on the cladding surface of the optical fiber, SEM images of (**c**) Optical fiber coated with cobalt oxide nanoparticles, (**d**) Micro-cavities formed on the cladding optical fiber.

## Materials and methods

### Synthesis of Co$_3$O$_4$ nanoparticles

To prepare Co$_3$O$_4$ nanoparticles, 2.95 g of cobalt acetate tetrahydrate ((CH$_3$COO)$_2$Co$\cdot$4H$_2$O) is dissolved in 20 mL of distilled water under continuous stirring to create a homogeneous precursor solution. Upon dissolution, it dissociates into its ionic constituents, releasing cobalt ions and acetate ions into the aqueous solution given as,

1 $$\begin{aligned} \text {Co}(\text {C}_{2}\text {H}_{3}\text {O}_{2})_{2}.\text {4H}_{2}\text {O (s)} \longrightarrow \text {Co}^{2+} \text {(aq)} + \text {2C}_{2}\text {H}_{3}\text {O}_{2}^- \text {(aq)} + \text {4H}_{2}\text {O (l)} \end{aligned}$$

Sodium hydroxide (NaOH) is gradually added dropwise to the solution and stirred constantly at 90$^0$C for one hour. This process facilitates the formation of cobalt hydroxide (Co(OH)$_2$) as a precipitate, which is then cooled to room temperature. The precipitate is transferred into a centrifuge tube and centrifuged at 7000 rpm for 20 minutes to separate it. In the first method, the precipitate is calcined in a muffle furnace at 350$^0$C for 3–5 hours in an air atmosphere^30^. The precipitation and calcination process is given by:

2 $$\begin{aligned} & Co(C_{2}H_{3}O_{2})_{2}.4H_{2}O (aq) + 2NaOH (aq) \rightarrow Co(OH)_{2} (s) + 2NaC_{2}H_{3}O_{2} (aq) + 4H_{2}O (l) \end{aligned}$$

3 $$\begin{aligned} & {6\text {Co(OH)}_{2} \text {(s)} \mathop {\longrightarrow }\limits _{900^\circ \text {C}}^{\Delta } 2\text {Co}_{3}\text {O}_{4} \text {(s)} + 6\text {H}_{2}\text {O (g)}} \end{aligned}$$

During this calcination process, cobalt hydroxide decomposes into cobalt oxide. Alternatively, in the second method, the centrifuged solution is placed in a ceramic container and allowed to dry naturally at room temperature for 15–20 days, during which cobalt acetate tetrahydrate gradually decomposes to form cobalt oxide nanoparticles as shown in Fig. 1. Both methods yield high-purity Co$_3$O$_4$ nanoparticles with desirable properties, such as stability and functionality.

#### Fourier transform infrared spectroscopy

Fourier Transform Infrared (FTIR) spectroscopy is initially applied to confirm the existence of the first-order chemical bonds in the synthesized Co$_{3}$O$_{4}$ nanoparticles^31^. Broad bands of absorption in the 2300–3700 cm^−1^ range indicated stretching vibrations of hydroxyl groups (–OH), indicating surface functional groups or adsorbed water. The presence of these hydroxyl groups makes their incorporation important because they can affect surface chemistry and increase the interaction of the acetone molecule, and hence contribute significantly to sensing performance^32^.

#### Dynamic light scattering

Dynamic Light Scattering (DLS) is employed to measure the hydrodynamic diameter and dispersity of the nanoparticle suspension^33^. In spite of some particle clustering, the intensity distribution is monomodal and indicates that most of the nanoparticles are within a size range of 300–500 nm. This dispersion profile indicates that although the primary nanoparticle size can be smaller, aggregation in water leads to an increased apparent hydrodynamic diameter. The integrity of the DLS data is additionally confirmed by the autocorrelation function, which indicates a high correlation coefficient, signifying minimal noise and a stable signal. Backed up by this, analyses of cumulants and the fit of the distribution reveal a fairly even trend in dispersion, corroborating the accuracy of the determined sizes of nanoparticles.

#### Scanning electron microscopy

SEM compares the fiber coatings obtained by the two functionalization processes. Under Method 1, where the fiber core is coated immediately after cladding removal, SEM images show irregular nanoparticle coverage, with obvious agglomerates and naked core areas. The thickness of the Co$_3$O$_4$ nanoparticle layer immobilized on the fiber sensing region is estimated to be in the nanometer-to-submicron range, as inferred from SEM surface morphology and comparative image analysis. Surface irregularity is due to insufficient surface roughness and poor adhesion on the untreated silica surface^34^.

**Fig. 3:**
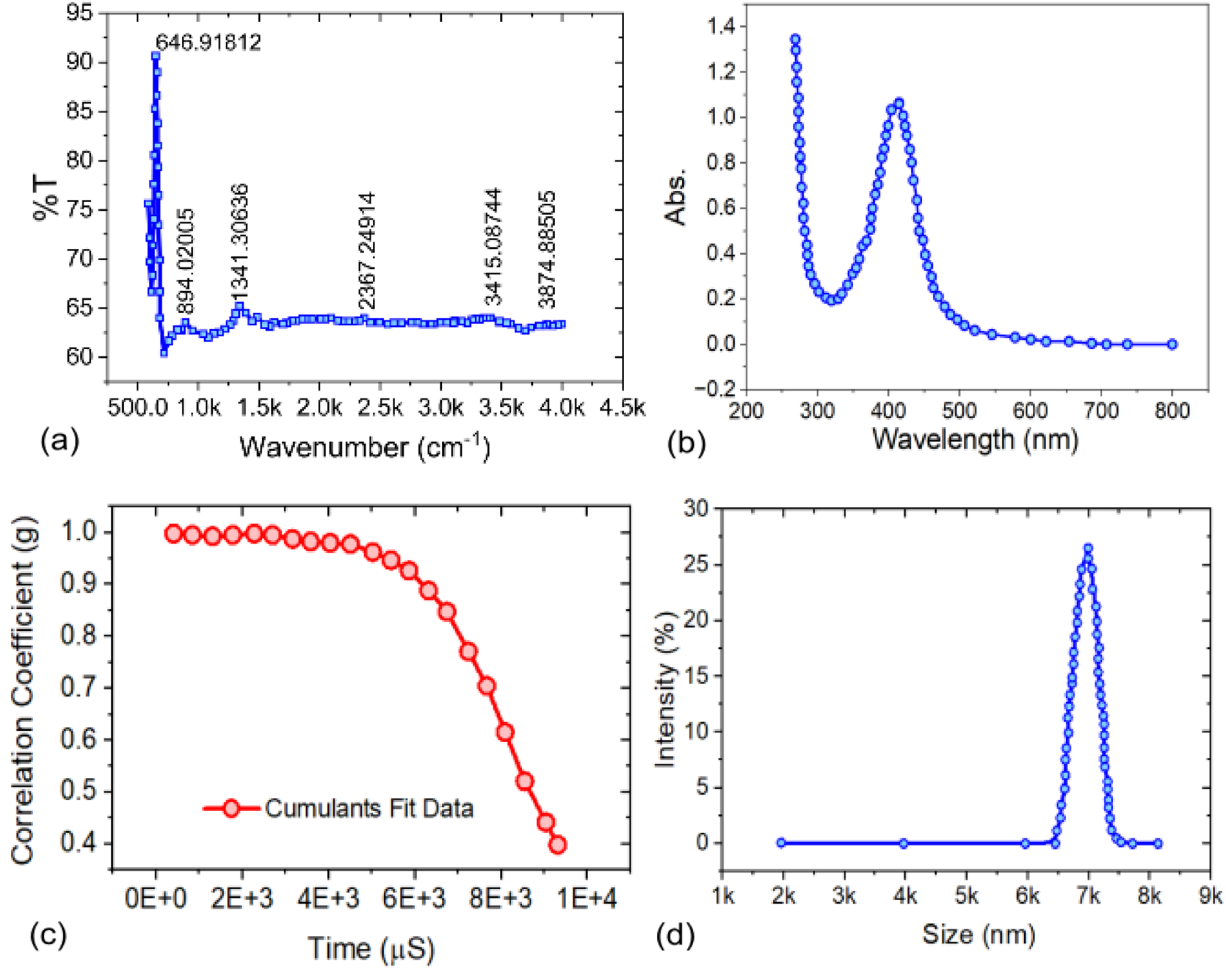
(**a**) FTIR spectrum of Co$_3$O$_4$ nanoparticles (**b**) UV-Vis absorption spectrum indicating strong electronic transitions in the spinel lattice, Dynamic Light Scattering (DLS) analysis of Co$_3$O$_4$ nanoparticles: (**c**) Autocorrelation function showing cumulants fit indicating high signal stability; (**d**) Size distribution graph showing dominant particle size range in terms of intensity.

#### Ultraviolet-visible spectroscopy

The optical properties of synthesized Co$_{3}$O$_{4}$ nanoparticle were studied using Ultraviolet-Visible (UV-Vis) spectroscopy. The UV–Vis absorption spectrum of the synthesized Co$_{3}$O$_{4}$ nanoparticles shows a prominent absorption peak centered at approximately 414 nm, which is attributed to ligand-to-metal charge transfer transitions characteristic of Co$_{3}$O$_{4}$. Initially, a wavelength of 650 nm was chosen due to the availability of a stable, low-cost optical source with high output stability and excellent compatibility with the optical couplers and photodetector used in the experimental setup. The spectrum of the synthesized nanoparticles shows a 414 nm peak resulting from an electronic transition within the spinel structure of the synthesized nanoparticles. Such nanoscale dimensions introduce quantum confinement effects and defect states, which are well known to shift the absorption edge of Co$_{3}$O$_{4}$ toward shorter wavelengths. The UV–Vis measurement is therefore used to confirm the semiconducting nature and optical activity of the nanoparticles rather than to define the sensor operating wavelength.

Ultraviolet-Visible (UV-Vis) spectroscopy is carried out to investigate the optical properties. The spectrum of absorption had a strong peak at approximately 414 nm and is due to electronic transitions from Co$^{2+}$ to Co$^{3+}$ ions in the spinel lattice. The transition plays a vital role in that it proves the semiconductor character of Co$_{3}$O$_{4}$ and is directly associated with its sensing mechanism. Upon interaction with the surface, the electronic environment may be altered, enabling shifts in the absorption spectrum for optical signal transduction. The peak also verifies that the synthesized nanoparticles have the energy band structure for optical interaction with volatile organic compounds (VOCs) such as acetone.

**Fig. 4:**
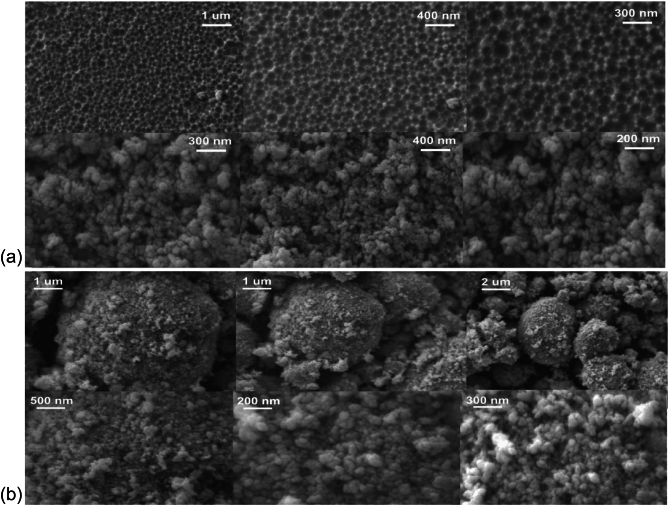
SEM results of (**a**) $Co_{3}O_{4}$ nanoparticles using the precipitation method at different magnifications and (**b**) micro-cavities formed and coating of $Co_{3}O_{4}$ nanoparticles on the cladding surface at different magnifications.

The characterization methods confirm that the Co$_{3}$O$_{4}$ nanoparticles prepared by the precipitation method possess suitable physical, chemical, and optical properties necessary for effective acetone detection. Their distinctive crystalline structure, porous and rough morphology, reasonable particle size, and characteristic electronic transitions all cumulatively justify their incorporation into optical fiber-based sensor platforms^35^.

### Single-mode fiber coating with Co$_3$O$_4$ nanoparticles

Acetone molecules do not possess intrinsic adsorption capability; instead, adsorption occurs through interaction with active sites present on the surface of Co$_3$O$_4$ nanoparticles. Co$_3$O$_4$ is a *p*-type semiconductor that provides oxygen vacancies and cobalt cationic sites (Co$^{2+}$/Co$^{3+}$), which facilitate adsorption of polar volatile organic compounds. Acetone molecules contain a polar carbonyl group (C=O), enabling dipole interactions with these active sites and leading to reversible adsorption on the nanoparticle layer. Deposition of the Co$_3$O$_4$ film on the optical fiber introduces additional optical attenuation due to absorption and scattering in the coating. This produces a constant reduction in transmitted intensity, which is recorded as the baseline signal during sensing measurements. This work utilizes a typical step-index single-mode fiber (SMF) with 9 $\mu$m core and 125 $\mu$m cladding diameter, obtained from Thorlabs, and a numerical aperture (NA) of 0.14. The fiber is cut into pieces 10 cm in length for convenience. All chemicals employed in the coating process such as Cobalt (II) Acetate Tetrahydrate (Co(CH$_{3}$COO)$_{2}\cdot$4H$_{2}$O, 0.3 M), Sodium Hydroxide (NaOH, 0.2 M), Hydrofluoric Acid (HF, 40%, AR grade), Nitric Acid (HNO$_{3}$, 70%, AR grade), acetone, and ethanol are sourced from SRL and Merck. All solutions are prepared using deionized water. Due to the handling of dangerous reagents like HF and HNO$_{3}$, all experiments are performed in a fume hood as per stringent safety guidelines. The nanoparticles are to be coated on the cladding surface of the optical fiber. The modification of the cladding surface has been done in two ways: (a) Manual cladding removal and (b) chemical Vapour Etching Method.

In the manual cladding removal process, individual segments of the fibers are initially cleaned by immersion in isopropyl alcohol and the polymer coating over the fiber is removed manually using acetone. A middle section of about 3–4 cm is then stripped of its cladding using a cleaver to expose the core as shown in Fig. 2(a) and immersed in a newly prepared suspension of Co$_{3}$O$_{4}$ nanoparticles. Sample is removed and dried at room temperature for 15–20 days^36^. However, the strength of the fiber gradually decreases, and unsuitable for low-concentration acetone sensing.

**Fig. 5:**
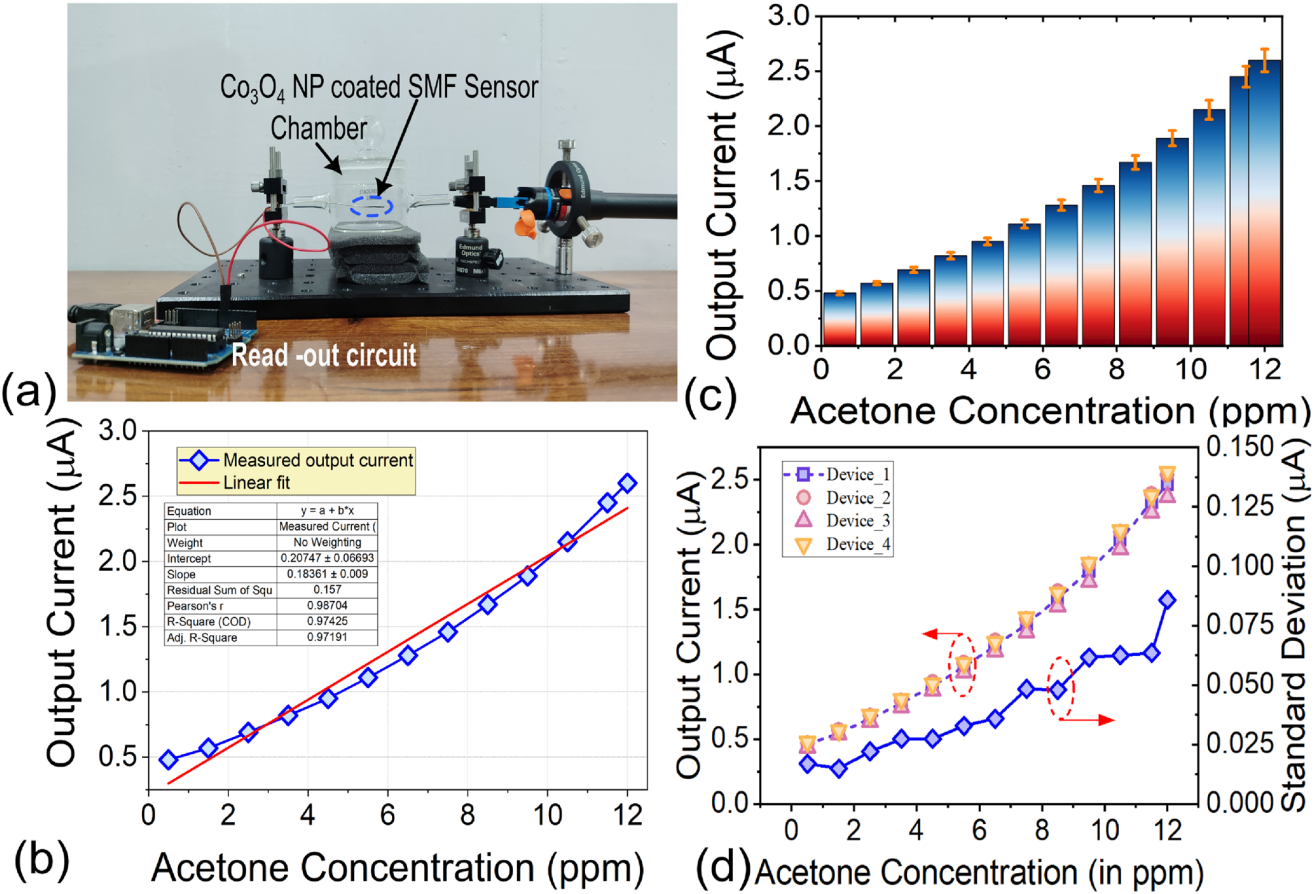
(**a**) Cobalt oxide nanoparticle (Co$_3$N$_4$) coated single-mode fiber sensor setup, (**b**,**c**) Output current for different acetone concentrations with linear and standard deviation, (**d**) Output current for different sensor for different acetone concentrations with standard deviation.

In this method, CVE-assisted dip-coating, the fiber is subjected to extra surface treatment before applying nanoparticles. After the removal of the jacket, the cladding area is inserted into a sealed Teflon chamber for Chemical Vapour Etching (CVE). This procedure exposes the cladding to acidic vapours produced from a 1:6 volume ratio mixture of hydrofluoric acid and nitric acid^37^. The etching is then carried out under a controlled temperature of 40$^{0}$C for 40 to 50 minutes as illustrated in Fig 2(b). The objective of CVE is to create microstructural roughness and porosity on the cladding surface, which increases nanoparticle adhesion and maintains the optical fiber’s strength without exposing the core. Following etching, the chamber is flushed, and the fiber is removed and cleaned.

After CVE treatment, the fiber is dip-coated with the same Co$_{3}$O$_{4}$ nanoparticle suspension and process mentioned in the first method. The coated fiber is dried under ambient conditions for 15–20 days to obtain stable film formation as shown in Fig. 2(c)(d).

The adhesion is checked by applying and removing a sticky tape onto the surface of the coated area, which is found to have minimal nanoparticles sticking to the tape. The effective sensing length of the Co$_{3}$O$_{4}$-coated optical fiber is approximately 3–4 cm, corresponding to the functionalized region exposed to acetone vapor. Increasing the sensing length enhances the interaction volume between the guided optical field and the analyte, thereby improving sensitivity. However, excessively long sensing regions may introduce higher propagation losses, increased noise, and reduced signal stability. Therefore, the selected sensing length represents an optimal balance between sensitivity enhancement and optical signal integrity. SEM examination of this top most coating offers comparative evidence regarding the quality and evenness of nanoparticle adhesion between the two procedures. A detailed comparision between two methods illustrated in Table.1.

**Table 1:** Comparison between two methods.

Feature/Parameter	Method 1: Dip-Coating (Manual Cladding Removal)	Method 2: CVE-Assisted Dip-Coating
Surface Preparation	Manual removal of cladding	Chemical Vapour Etching (CVE) using HF/HNO$_3$ vapors
Exposure of Core	Yes (3–4 cm exposed)	No (cladding modified, core intact)
Nanoparticle Adhesion	Poor, uneven, with agglomerates	Strong, uniform, minimal agglomeration
Mechanical Integrity	Reduced due to core exposure	Maintained due to intact core
Coating Uniformity (SEM Observation)	Irregular, patchy	Homogeneous, well-distributed
Sensor Stability	Lower	Higher
Suitability for Real-Time Sensing	Limited	High

## Results and discussion

The FTIR spectrum presented intensive absorption bands between 600 and 700 cm$^{-1}$, which are Co–O stretching vibrations typical for the spinel-phase cobalt oxide formation. Apart from this, the band around 1632 cm$^{-1}$ is ascribed to bending vibrations of adsorbed water molecules, indicating hydration of particle surfaces as shown in Fig. 3(a). UV-Vis spectroscopy identifies a strong absorption peak at 414 nm, corresponding to Co$^{3+}$ $\rightarrow$ Co$^{2+}$ electronic transitions in the spinel lattice as shown in Fig 3(b). This indicates that the material is semiconductor in nature and capable of optically reacting to external stimuli like volatile organic molecules. In the DLS analysis, a high correlation coefficient from the autocorrelation function, complemented with supporting fit analyses, assures proper size measurement and a consistent synthesis process, as shown in Fig 3(c). This suggests moderate dispersion with some agglomeration, but a largely monomodal distribution indicating synthesis uniformity. DLS measurements show a Z-Average particle size of 511.2 nm and a PDI of 0.589 (Fig 3(d)).

Scanning Electron Microscopy (SEM) gives a close-up view of the surface microstructure and morphology of the Co$_{3}$O$_{4}$ nanoparticles synthesized and the optical fiber coating, as shown in Fig 4. At lower magnifications ($\sim$9.25K$\times$), the SEM images show irregular, porous agglomerates between 2–5 $\mu$m, whereas at higher magnifications (up to 190.55K$\times$), densely packed crystallites with a grain size of 20–50 nm are visible. These crystallites exhibit a cauliflower-like morphology, forming larger spherical agglomerates with diameters of 1–2 $\mu$m, which display a hierarchical nanostructure that is favorable for gas adsorption.

Comparatively, the sample obtained with the CVE method greatly improved surface morphology. SEM micrographs of the CVE-treated core indicate a porous, rough texture formed due to acid vapour interaction, promoting better nanoparticle attachment. Following dip-coating on this altered surface leads to a homogeneous, well-adhered layer of nanoparticles with little agglomeration and better distribution than Method 1, as shown in Fig. 4(b).

In Fig. 5(a), the sensor system setup is illustrated, which uses a fault locator as the light source, emitting at 650 nm, to provide a stable optical beam for acetone sensing. When acetone molecules are adsorbed onto the Co$_3$O$_4$ nanoparticle layer, the optical properties of the coating are modified. Since the sensing experiments are conducted at room temperature, the interaction mechanism is dominated by reversible adsorption rather than catalytic oxidation typically observed in high-temperature chemoresistive sensors. The adsorption of acetone molecules alters the optical absorption coefficient of the Co$_3$O$_4$ coating, thereby affecting light propagation through the fiber. This results in a change in the transmitted optical intensity, which is detected by the photodiode and converted into an electrical signal used to determine the sensor response. The acetone sensing mechanism does not rely on direct spectral overlap with the UV–Vis absorption peak of $Co_{3}O_{4}$; instead, acetone adsorption modulates light transmission through surface absorption and scattering effects, enabling effective sensing at 650 nm. The light is coupled into a 125 $\mu$m core optical fiber, coated with cobalt oxide ($Co_{3}O_{4}$) nanoparticles to enhance sensitivity. An optical coupler injects light into the fiber, while another coupler directs the transmitted signal to a photodiode detector, which converts the optical signal into an electrical one, reflecting intensity changes due to acetone interaction with the fiber coating. The photodiode’s electrical signal is sent to a microcontroller (Arduino/ESP-32) for signal conditioning and processing. The data is then transmitted to a mobile app, displaying real-time acetone concentration. The nanoparticle-coated fiber section is housed in a sealed glass chamber to control acetone vapor exposure, minimizing environmental interference and ensuring accurate, repeatable measurements. The operational reliability of the sensor was evaluated under ambient conditions through repeated acetone sensing cycles. Stable and reproducible responses were observed for five consecutive measurement cycles, after which a gradual degradation in performance occurred, likely due to acetone adsorption, humidity effects, surface contamination, and agglomeration of $Co_{3}O_{4}$ nanoparticles.

The output current was 0.48 $\mu$A for 0.5 ppm acetone and changed to 2.6 $\mu$A for 12 ppm (Fig. 5(b)). The sensitivity of the $Co_{3}O_{4}$ coated SMF sensor was given by $S_e=\frac{\Delta ~I_D}{\Delta ~C_A}$, where $\Delta ~I_D$ and $\Delta ~C_A$ are the change in current and acetone concentration, respectively. The sensor response for different acetone concentrations ($C_A$) is depicted in Fig.5(b). The sensitivity is found to be 0.18 $\mu$A/ppm and the root mean square error (RMSE) of the linear fit was used to quantify non-linearity which was $\sim$3%. The limit of detection (LOD) was estimated using a signal-to-noise approach and is approximately 0.15 ppm, based on three times the baseline noise. Error bars in Fig. 5(c) represent the standard deviation obtained from five repeated measurements at each acetone concentration. Batch-to-batch reproducibility of the proposed sensor was examined by repeating the fabrication process using the same nanoparticle synthesis and CVE-assisted coating procedure. Multiple sensors were independently prepared and tested under identical experimental conditions. The statistical variation in the measured photodiode current was analyzed, and the standard deviation of the response was found to be approximately $\sim 4\%$. Fig. 5(d) depicts different sensor outputs and standard deviations obtained from measurements, indicating good reproducibility and reliability of the fabrication process. The proposed sensor is compared with other works in Table 2. Selectivity is an important parameter for practical acetone sensing, particularly in the presence of other VOCs. Although systematic cross-sensitivity experiments with interfering VOCs (e.g., ethanol, methanol, and isopropanol) were not performed in this study due to experimental constraints, the sensing behavior can be explained by the intrinsic properties of $Co_{3}O_{4}$. As a p-type semiconductor with abundant surface oxygen species and catalytic activity, $Co_{3}O_{4}$ exhibits stronger interaction with carbonyl-containing molecules such as acetone compared to alcohol-based VOCs, leading to enhanced charge transfer and optical modulation.

**Table 2:** Comparison with other acetone sensors.

Refs.	Method	Temp. ($^0$C)	Sensitivity $\mu$A/ppm	LOD (ppm)	Complexity
^38^	8-layer $ReS_2$-FET	RT	0.025	NA	High, Complex System
^39^	$WO_3$ Nanosheets	350	0.0125	1.54	Moderate
^39^	$WS_2$ Nanosheets	RT	0.16	1.54	Moderate
^40^	NiO-ZnO	260	0.0325	5	Moderate
This work	Co$_{3}$O$_{4}$ coated SMF	RT	0.18	0.15	Low cost, Simple

## Conclusion

The research successfully demonstrated fiber-optic acetone Sensor using a single mode silica fiber (SMF) coated with cobalt oxide (Co$_{3}$O$_{4}$) synthesized through a controlled chemical vapor etching (CVE) pretreatment process. The etching was carried out using a 1:6 HF/HNO$_3$ vapor mixture at 40$^0$C for 40–50 minutes, which effectively enhanced the fiber surface roughness and adhesion characteristics for subsequent coating. The improvement in adhesion of coatings after CVE treatment was investigated using SEM images and a qualitative test of tape adhesion. Quantitative tests of thickness, surface roughness, and adhesion strength were not carried out in this study because of instrumental limitations; they will be considered in future work. The dip-coated and annealed Co$_{3}$O$_{4}$ layer exhibited stable surface morphology and strong interaction with acetone molecules, enabling an efficient modulation of optical intensity. A practical sensing setup was developed wherein one optical coupler was used for light injection into the functionalized fiber, while another directed the transmitted optical signal to a photodiode detector. The detector converted the modulated optical signal into an electrical response, which was further processed using a microcontroller (Arduino/ESP-32) for signal conditioning and quantitative evaluation. The sensor exhibited a measurable and reversible response to acetone vapor, with the output current varying from 0.48 $\mu$A at 0.5 ppm to 2.6 $\mu$A at 12 ppm acetone concentration. The sensitivity of the device was determined to be 0.18 $\mu$A/ppm with a non-linearity of approximately 3%. These results confirm that the CVE-assisted Co$_{3}$O$_{4}$ coating method significantly improves the fiber’s surface activity and optical transduction capability. The fabricated sensor thus demonstrates potential for deployment as a low-cost, miniaturized, and high-performance volatile organic compound (VOC) sensing platform. Future work can focus on extending this approach toward selective detection of other VOCs, comprehensive cross-sensitivity, interference studies, and improving long-term sensor reliability, integration with wireless data acquisition systems, and optimization of coating thickness for enhanced response and recovery times.

## Data Availability

The datasets used and/or analysed during the current study available from the corresponding author on reasonable request.
